# The Functionally Grading Elastic and Viscoelastic Properties of the Body Region of the Knee Meniscus

**DOI:** 10.1007/s10439-021-02792-1

**Published:** 2021-06-01

**Authors:** Jared Maritz, Greta Agustoni, Kalin Dragnevski, Stéphane P. A. Bordas, Olga Barrera

**Affiliations:** 1 University of Oxford, Oxford, UK; 2 Department of Health Science and Technologies, ETH, Zurich, Switzerland; 3 Institute of Computational Engineering Sciences, University of Luxembourg, Luxembourg, Luxembourg; 4 Institute of Research and Development, Duy Tan University, K7/25 Quang Trung, Danang, Vietnam; 5 Cardiff University, School of Engineering, Cardiff, UK; 6 School of Engineering, Computing and Mathematics, Oxford Brookes University, Wheatley Campus, Oxford, OX33 1HX UK; 7 Department of Medical Research, China Medical University Hospital, China Medical University, Taichung, Taiwan

**Keywords:** Knee meniscus, Grading mechanical properties, Mechanical testing

## Abstract

The knee meniscus is a highly porous structure which exhibits a grading architecture through the depth of the tissue. The superficial layers on both femoral and tibial sides are constituted by a fine mesh of randomly distributed collagen fibers while the internal layer is constituted by a network of collagen channels of a mean size of 22.14 $\mu$m aligned at a $30^{\circ }$ inclination with respect to the vertical. Horizontal dog-bone samples extracted from different depths of the tissue were mechanically tested in uniaxial tension to examine the variation of elastic and viscoelastic properties across the meniscus. The tests show that a random alignment of the collagen fibers in the superficial layers leads to stiffer mechanical responses (*E* = 105 and 189 MPa) in comparison to the internal regions (*E* = 34 MPa). All regions exhibit two modes of relaxation at a constant strain ($\tau _1 = 6.4$ to 7.7 s, $\tau _2$ = 49.9 to 59.7 s).

## Introduction

The menisci play an important role in the knee joint by transmitting loads between the femur and tibia and providing structural stability and shock absorption.^17^ Previous studies on meniscal architecture have highlighted changing arrangements and orientations of collagen fibres across the depth of the tissue. The superficial layers of the meniscus (in contact with tibial and femoral cartilage surfaces) exhibit a randomly distributed mesh of thin collagen fibrils while the internal layers contain collagen fibers running along the circumferential direction, thus dictating the load bearing capacity of the meniscus.^22^ A more detailed study has revealed further insight on the dimensions and arrangements of the collagen fibers in both the circumferential and the radial directions in the internal regions of the tissue. It has been noted that collagen fibrils in the radial direction (radial tie fibers) form a honeycomb-like network containing other collagen fibrils (diameter of 5 $\mu$m) tightly packed together running in the circumferential direction.^24^ It is fundamental to note that these results are limited by the artefacts introduced by the sample preparation such as fixation, mechanical and chemical peeling and dehydration.

Vetri *et al*.^29^ analysed the micro- and nanoscale architecture of human menisci without introducing artefacts. Fresh and untreated human meniscal samples were observed with multiphoton microscopy and environmental scanning electron microscopy. In their work a three dimensional structure of collagen bundles arranged in “honeycomb-like” cells is observed, reflecting earlier findings.^24^ Furthermore, the authors detected “honeycomb-like” compartments of sizes varying from micro (25 to 100 $\mu$m) to macro (600 $\mu$m to 1 mm) scales. Furthermore, each honeycomb compartment was discovered to contain pores, prompting further research using micro computed tomography.^2^ This research created 3D reconstructions, showing the pores as a network of collagen channels with mean diameter 22.14 $\mu$m, aligned at a $30^{\circ }$ inclination with respect to the vertical direction. In the superficial regions, it was found that such channels were aligned randomly, with the tibial superficial region larger than the femoral.

Knowledge of the structure of the collagen fibres and channels aids in the understanding of meniscal mechanics. During *in-vivo* loading, the meniscus experiences a large, vertical compressive force. The fixed meniscal horns gives rise to circular traction, leading to a tensile force in the circumferential direction. The fluid flowing through the channels is believed to be responsible for the time dependent behaviour of the tissue. The pore pressure is mainly responsible for the load bearing capacity of the tissue.

Nonlinear mechanical properties of articular cartilage have been measured by the means of indentation testing.^8^ Innovative inverse techniques have been adopted to evaluate the variation of mechanical parameters in tissues.^11^

**Figure 1:**
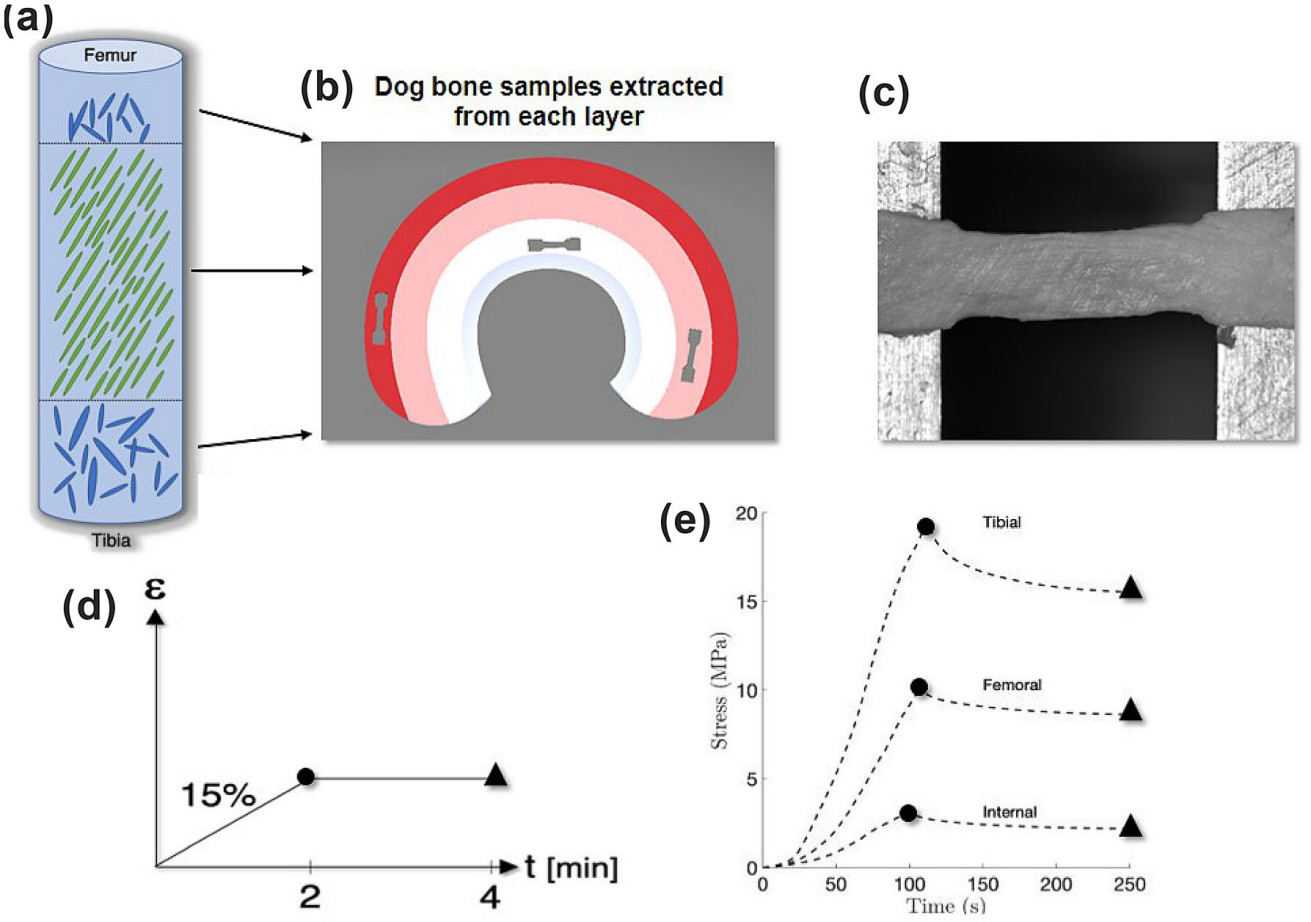
Study overview. (a) Schematic representation of a cylindrical sample extracted from the body region of the meniscus, the superficial layers (femur and tibia sides) show randomly distributed collagen fibers, the internal layers are constituted by collagen channels oriented at 30° from the vertical^2^; (b) Depiction showing dog-bone samples taken from a meniscal layer, samples were extracted from all three layers (superficial femur, tibia and internal layer; (c) Image of a sample pre-testing; (d) Details of strain-controlled uniaxial testing; (e) Average stress-time results from the three layers (femoral, tibial and internal) in the body region. Shown are the averaged curves of several tested samples for each region.

The stress–strain curve is sigmoidal in shape, beginning with a low stiffness toe region of fibre recruitment, followed by a stiffening as strain increases, before a gradual softening and eventual fracture.^21^ The yield point has been defined as the transition from the high stiffness to strain-softening region. Studies define a quasi-linear region at the yield point, where the slope is greatest, and use this to calculate an elastic modulus.^7^,^13^,^14^,^23^,^26^ Moduli found for the various regions have large differences between studies, with results of 5 ± 1.22 to 198.4 ± 87.5 MPa for samples taken from similar regions.^13^,^23^ The large differences and uncertainties reported are due to a combination of the inherent variance of biological materials as well as differences in methods. Modulus calculation methods can affect results,^21^ while sample sizes and shapes can change fibre orientations or cause sectioned fibres that lower tensile strength.^14^ Despite the large variations in values, a common trend seen is that superficial regions of the meniscus exhibit a stiffer response than deeper sections. Comparisons between anterior and posterior horns and the central body are less evident, although the horns have been reported to be stiffer. This work presents extensive mechanical testing results performed following a robust and repeatable procedure for both extracting samples^6^ and executing the experiments^16^ for all of the porcine menisci analysed.

The biphasic nature of the meniscus introduces large time-dependent behaviour that provides the meniscus with its shock absorbing properties. This has been well studied in compression, where it is thought that fluid pressurisation and frictional drag govern behaviour.^9^,^18^ In tension, this behaviour is generally ignored as low strain rates are used, and fluid caused viscosity is thought to be negligible. Fibrous tissues are, however, known to exhibit viscous effects such as relaxation, and thus have strain-rate dependent effects independent of fluid-caused viscosity. One study investigated this behaviour, although it was performed at a time when knowledge of the meniscal microstructure was limited.^27^ It was found that there was a slight but significant relationship, with the elastic modulus increasing slightly with strain rate. Relaxation tests were also performed, where it was found that relaxation depended on the strain history, evidencing non-linear viscoelastic behaviour.^27^ A more recent study investigated strain-rate effects in articular knee cartilage, measuring strain rates from 0.1 to 80% per second.^4^ It was found that as strain rate increased, peak stress and modulus increased non-linearly. While these changes were less evident at high strain rates above 25% per second, at low strain rates changes were quite large. This indicates the strain rates could have an impact on the above meniscal results that have not been considered. The study attributed this strain-rate variance to collagen fibres as opposed to fluid effects, however, it did not make any suggestions as to the properties of the fibres that cause such an effect. It is also unclear how transferable the findings are from articular cartilage to meniscus. One other study investigated the relaxation of the meniscus, using their findings to calculate permeability coefficients.^15^ However, the stress–strain curve was treated as linear and the effects of the long strain ramping, which used a slow strain rate of $0.0001 \hbox { s}^{-1}$, were not included in the permeability parametrisation. It is evident that there is a need for viscoelastic data to accurately describe the tensile behaviour of the meniscus in the circumferential direction.

In summary, mechanical investigations of the meniscus are often limited and do not consider changes across regions of the meniscus or viscoelastic effects. In this study, uniaxial tensile mechanical testing is performed in regions across the meniscus, as shown qualitatively in the overview of the study in Fig. 1. Results can be linked to the recently discovered architecture in the body region.^2^

## Materials and Methods

**Figure 2:**
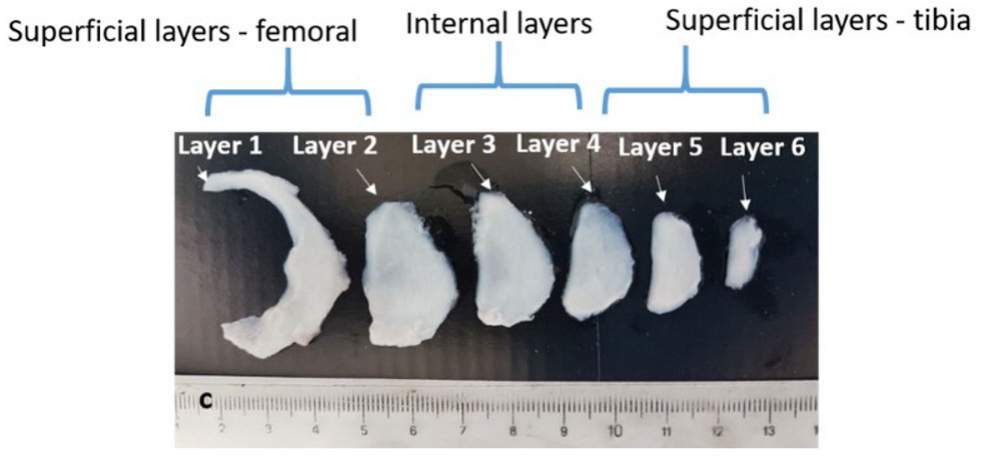
Results of the slicing process. It is possible to produce six to ten slices for each meniscus and differentiate between superficial and internal layers which have different mechanical behaviours.^6^

Testing was performed using a total of 9 porcine medial menisci. Each menisci was placed in a cavity in a block of extruded polystyrene and covered with Polyfreeze (Sigma-Aldrich, United States). These were then frozen by submersion in liquid nitrogen and sliced using an MBS 240/E Micro Band Saw (Proxxon, Germany). The blade used had 14-teeth-per-inch and was 0.4 mm thick, set to a speed of approximately 250 m/min. The menisci were sliced normal to the vertical axis, from the tibial side to the femoral side, producing slices with thicknesses of 0.7 to 1.1 mm as in Fig. 2. This procedure has been found not to alter microstructural properties.^6^ Following this, dog bone samples were stamped and tested in uniaxial tension using a previously developed method.^16^ Dog bone samples were taken at various vertical and radial depths. In total, 8 samples were tested from the internal region, 5 from the femoral layer and 2 from the tibial layer. To be considered as originating from a tibial or femoral superficial layer, the sample would have come from within 30% of the total meniscal depth to the respective surface of the meniscus. It is important to note that these depth classifications are much thicker than the actual superficial layers. These larger region definitions were used due to the difficulty of taking samples from very close to the surface. These layers are thus more indicative of a transitional region between the actual superficial layers and the internal layer.

A speckle was applied to sample surfaces using black ink and an air brush and imaged during testing to enable DIC strain analysis. Specimens were mounted in a 200 N micro tensile stage (Deben UK ltd) and kept hydrated throughout the test using a Phosphate Buffered Saline (PBS) solution. The solution was only applied to the samples from the bottom surface to ensure the speckle was not distorted. Samples were clamped with a length of 10 mm between grips. The clamps had a serrated edge to grip the large surface area at the ends of the dog bone samples, which prevented slipping. Displacement controlled experiments were then carried out at a displacement rate of 1 mm/min (a strain rate of $0.00167 \hbox { s}^{-1}$) to a total distance of 12 mm. Due to the introduction of slack in the samples during clamping, loading onset was delayed in each sample by a variable amount. 0% strain was taken for each experiment at the point where the force-displacement curve passed a threshold of 0.02 N. This meant that at full extension (12 mm), strain was typically near 15%. This strain was then held constant for 2 min to examine stress relaxation behaviour.

Engineering stress was calculated using the force readings from the tensile stage and the initial cross sectional area of the samples. The sample dimensions were found using vernier calipers. Strain was found using DIC processing, performed with the software DaVis (LaVision). As strain was more uniform in the central regions of the sample, and less uniform near the clamps, the software was used to find the strain across an initial 5 mm displacement in the centre of each sample.

From the stress–strain curves, numerical results were calculated for elastic moduli, yield stresses and yield strains for each test. The yield stresses and strains were calculated at the point of inflection in each stress–strain curve,^21^ with the calculated modulus of each sample being the tangent at this yield point.

Stress-time curves were analysed by fitting a generalised Maxwell model to the relaxation portion of each curve using non-linear least squares regression. When fitting the model it was found that a one-element model did not reflect behaviour well for most samples, and so a two-element model was used of the form of Eq. (1). In this model, $\sigma (t)$ is the measured stress, $\epsilon$ is the constant strain of the sample during relaxation, $E_{r1}$ and $E_{r2}$ are the relaxation moduli for the two modes of relaxation, and $\tau _1$ and $\tau _2$ the respective relaxation times, while $\sigma _\infty$ is the stress at $t=\infty$. This model assumes the stress was applied as a step change, for simplicity. This means, however, that the relaxation that occurs during loading is not accounted for in this model.

1 $$\begin{aligned} \frac{\sigma (t)}{\epsilon }=E_{r1}e^{-t/\tau _1}+E_{r2}e^{-t/\tau _2}+\frac{\sigma _\infty }{\epsilon } \end{aligned}$$

## Results

### Stress–Strain Results

**Figure 3:**
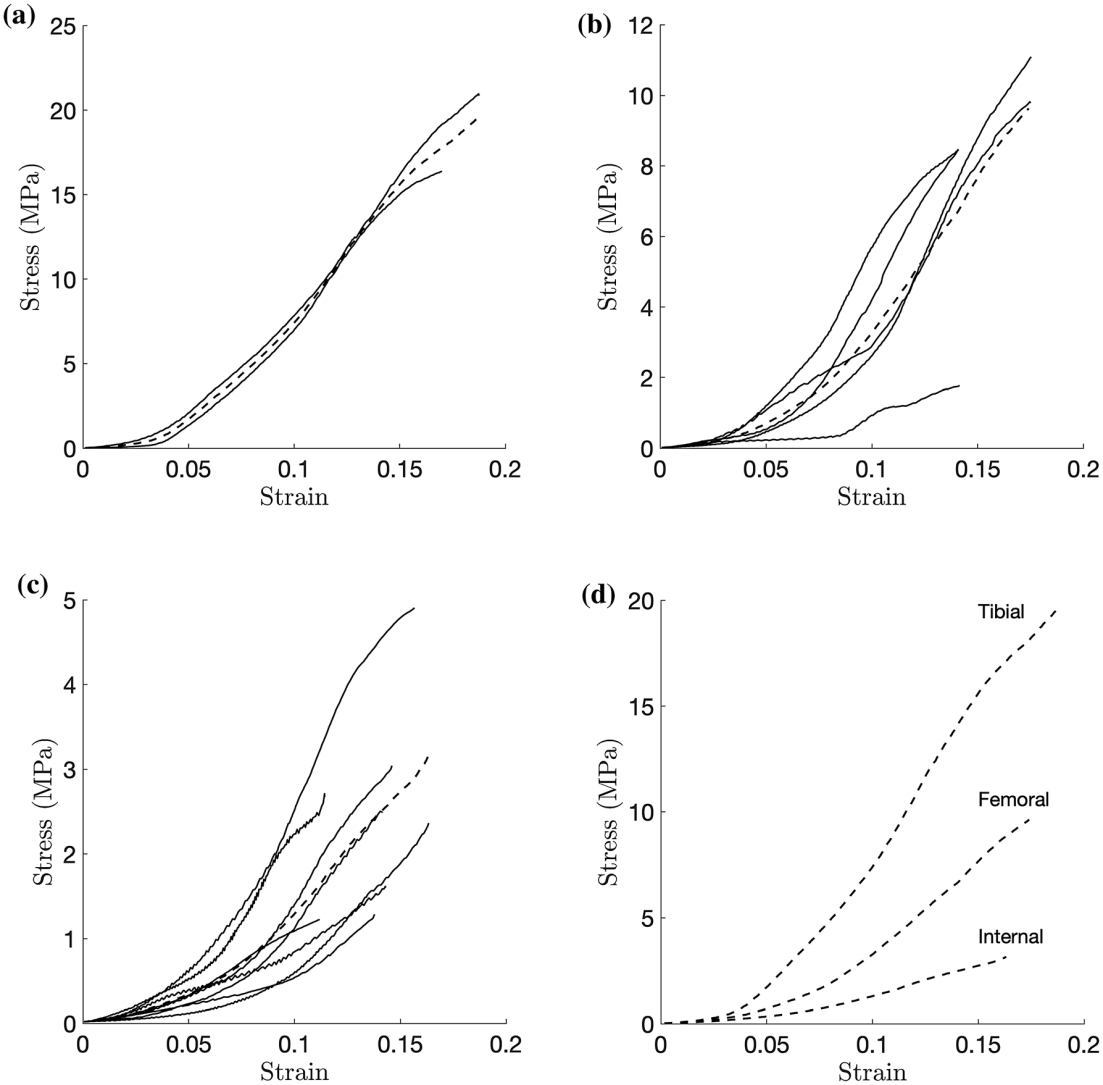
Stress vs. strain curves of the central body, with averages (dashed lines). (a) Tibial Layer; (b) Femoral Layer; (c) Internal Layer; (d) Averages.

Results are shown grouped by the vertical regions (tibial layer, femoral layer or internal layer) from which the samples were taken. An average curve was produced for each grouping to provide a rough visualisation for each region. This was calculated by initially finding the average stress at each strain point. As the tests ended at varying strains, segments of the averaged curve were then vertically shifted to eliminate the sharp jumps that occurred due to this staggered termination of curves. These curves are thus not perfectly smooth and should be used only as a quick comparison between regions. The elastic modulus, yield stress and yield strain for each region reported in Table 1 are the average of the values calculated for each individual test in the region (Fig. 3).

**Table 1:** Elastic moduli at yield point, yield stresses and strains (mean ± standard deviation).

Region	Elastic modulus (MPa)	Yield stress (MPa)	Yield strain
Central body tibial	189 ± 9	12.3 ± 3.2	0.13 ± 0.02
Central body femoral	105 ± 40	5.0 ± 2.3	0.12 ± 0.02
Central body internal	34 ± 15	1.7 ± 0.8	0.12 ± 0.02

### Stress-Time Results

**Figure 4:**
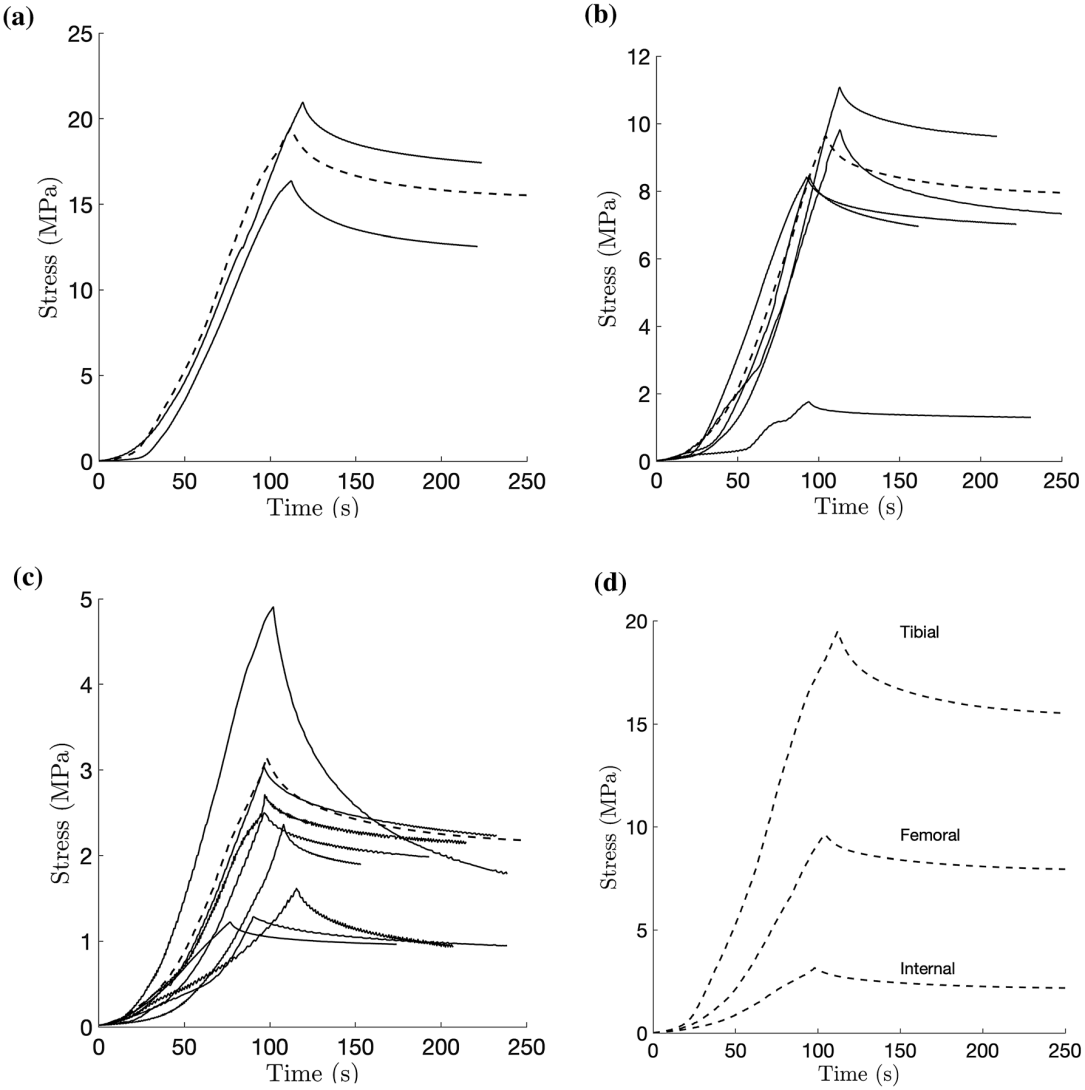
Stress vs. time curves of the central body, with averages (dashed lines). (a) Tibial Layer; (b) Femoral Layer; (c) Internal Layer; (d) Averages.

Stress-time results are shown for the same regions as above. The relaxation moduli and times, which are independent of the strain at which relaxation occurs, were averaged for each region and are reported in Table 2. An average curve was also produced for each region to enable approximate visual comparisons. The strain-increasing section of the average curve uses the average stress found in the stress–strain curves. The time data used for the average is calculated from the strain data, assuming the constant strain rate of $0.00167 \hbox { s}^{-1}$. An average relaxation curve was then produced using the found parameters and the stress and strain at the end of each averaged curve (Fig. 4).

**Table 2:** Viscoelastic relaxation parameters (mean ± standard deviation).

Region	$E_{r1}$ (MPa)	$\tau _1$ (s)	$E_{r2}$ (MPa)	$\tau _2$ (s)
Central body tibial	7.2 ± 0.8	7.2 ± 0.7	15.0 ± 1.9	49.9 ± 3.4
Central body femoral	3.5 ± 1.5	6.4 ± 1.8	6.9 ± 3.2	58.0 ± 13.0
Central body internal	1.9 ± 2.0	7.7 ± 4.0	4.5 ± 4.1	59.7 ± 23.6

## Discussion

### Stress–Strain Discussion

It is clear that the curves follow the expected sigmoidal shape shown in other studies. This involves the low strain ’toe’ region that involves fibre recruitment followed by a steeper, more linear section.

A previous study found Young’s modulus in bovine meniscus to be 198.4 MPa in the circumferential direction near to the surface, and 139.0 MPa in the deep zone.^23^ These results are similar to those found in this study of 189 and 105 MPa for the areas nearer the tibial and femoral surfaces respectively, although the deep zone modulus of 34 MPa is much lower. These differences may arise due to differences in the properties of bovine and porcine meniscus. A study that used porcine menisci found moduli in the central medial meniscus to be 94.54, 77.95 and 57.97 MPa for the tibial, femoral and interior regions respectively.^26^ The trend closely follows that found in this study, although the differences in values are much less. Further, another study using porcine menisci found Young’s moduli of 5.0 MPa for the medial central, with regions closer to the tibial and femoral surfaces exhibiting moduli of 8.6 and 12.7 respectively.^13^ It can be seen that there is much variation in the magnitudes of modulus values previously found, and those found in this study fall into this range. One study that investigated the yield stress and strain of the central, interior region found the yield stresses of 12–14 MPa and yield strains of 0.09 to 0.12.^21^ The yield strains are similar to those found in this study, and the higher yield stresses found may again be possibly explained by the use of bovine menisci in the cited study. All the differences summarised here may result from variation in sample preparation techniques, as the referenced studies use slicing techniques that may produce inconsistent and inaccurate samples that are avoided with the technique used in this study.^6^

In most curves, the post-yield section with strain softening is also visible. Looking at the results, distinct mechanical differences can be seen between the layers. The superficial layers are both stiffer than the internal layer. This can be explained by the differences in microstructure found above, with the randomly aligned collagen channels in the superficial layers exhibiting stiffer properties in order to withstand high contact forces. It is also worth noting that the tibial layer exhibits a stiffer response than the femoral layer, which can be explained by the comparatively larger thickness of the tibial layer.^2^ This causes the architecture of samples from near the tibial surface to more closely resemble the superficial architecture, compared to the femoral side which has a much thinner superficial layer and thus the samples more closely resemble the internal architecture.

While performing the research, an equal number of samples were taken across red, red-white and white regions from a range of depths. When processing results it was found that the much stronger correlation in behaviour was when samples were grouped by vertical depth instead of radial region. This means that in each grouping above, samples were from various radial zones. This may have introduced some variability in the behaviour, as well as causing an unequal number of samples for each region. This is most noticeable in the tibial layer from the central body, where only two samples were taken. Additionally, as previously mentioned, the superficial zone classifications for samples were much larger than the actual zones, and thus more indicative of a transitional region. While there are clear differences in behaviour between these transitional superficial regions and the internal layer, the behaviour inside the superficial layers are likely to be a more extreme case of the transitional regions investigated here, with even greater stiffness.

It is known that the elastic modulus is dependant on strain rate in soft tissues, with the modulus increasing with strain rate.^20^ Thus, the elastic moduli results found here are only accurate for strain rates similar to that used in this study, and can be expected to be higher for quicker strain rates. As many of the strain rates experienced by the meniscus *in vivo* are orders of magnitude higher than that used here, it would be of interest to repeat this experiment using higher strain rates.

One consideration that must be made is the low number of samples in the tibial layer, which may cause the results to not be a true reflection of the region. It is thus recommended that more data be retrieved for the central body tibial layer to confirm the much larger modulus in this region. Yield strains appear to be similar in value throughout the meniscus.

### Stress-Time Discussion

The need to use a two-element generalised Maxwell model highlights the existence of two modes of relaxation, each with its own calculated relaxation modulus and relaxation time. The magnitude of the relaxation moduli tends to follow the same trends as the elastic moduli, with regions of higher stiffness relaxing by a larger amount. The first mode had relaxation times of 6.4 to 7.7 s, while the second mode was 49.9 to 59.7 s. This shape is typical of soft tissues, which normally involve one quick and one long mode of relaxation. One explanation for the slow decay is due to a sequence of micro-yield occurrences. As one micro-structure yields, it passes the stress it was sustaining to another region until it also yields, with this process occurring slowly for very long periods of time.^5^ This appears to be in addition to the quick, sharper decay. While this quicker mode of relaxation may solely be caused by individual collagen fibres relaxing, the passage of fluid through the channels may also contribute. It has previously been thought that fluid-caused viscous effects are negligible at the strain rate used in this experiment, based on the understanding of a spaghetti-like collagen fibre architecture. It is unclear what effects the findings of collagen channels has on fluid-caused viscosity, and there is potential to find additional, quicker modes of relaxation if testing at much faster strain rates is done. Additionally, performing in situ ESEM relaxation tests would provide insight into the exact methods of decay and their interactions.

The relaxation moduli of the first, shorter mode was typically about half that of the second, longer mode in each region. It is expected that in reality, the moduli for the first mode is larger than recorded here. This is because the strain application was considered as a step change for this model. During the period of increasing strain, relaxation would have been occurring already and is not accounted for from looking at the relaxation section alone, thus giving rise to results that show a lower relaxation modulus. While the same effect would be true for the longer mode, it would have a much smaller effect due to the longer time period of relaxation. This strain step-change assumption means that the modelled relaxation is only truly applicable in situations where a similar strain rate is used to the experiment. A more detailed investigation into how relaxation changes with strain rates is thus recommended.

The relaxation data found here could be paired with the modelling of the stress–strain curve to produce a quasi-linear viscoelastic model that fully describes the tensile behaviour.^10^ Exponential models have been found to fit well to the early parts of the stress–strain curve.^19^,^25^,^28^ This could be done in addition with a modelling technique that simultaneously fits the ramping and relaxation data to the model, which eliminates the need for a step-change assumption when modelling relaxation.^1^

Another potential impacting factor is that these tests took the samples to post yield before the relaxation was observed, so it may not be an accurate representation of pre-yield relaxation. However, the tests did not go far beyond the yield strains, and thus the data can provide an approximation of pre-yield relaxation. Additionally, no pre-conditioning was performed on the samples, which may cause a change in mechanical properties.

Furthermore, a number of works in the literature (i.e. Refs. 3,12) show that preservation methods (mainly fresh freezing and cryopreservation) have an influence on the biomechanical properties of human lateral menisci. It has been reported that the elastic modulus and the point of rupture (UTS) are higher for the samples that were cryopreserved with respect to the fresh-frozen ones. It is therefore fundamental to pay attention to the preservation methods adopted when comparing experimental results reported in different studies.

To conclude, the variation in microstructure described by Agustoni *et al*.^2^ explains observed differences in mechanical behaviour between superficial and internal regions in the central body of the meniscus. The elastic moduli for the superficial regions (105 and 189 MPa) were much higher than those of the internal region 34 MPa). Relaxation results showed a quick decay ($\tau _1 = 6.4$ to 7.7 s) followed by a longer, slower one ($\tau _2 = 49.9$ to 59.7 s), indicating two modes of relaxation. The quicker mode is likely from fluid movement through the discovered channels, while the longer decay is from the relaxation of the collagen fibres. It is vital that these properties are taken into consideration during the design of synthetic implants to enable them to correctly mimic meniscal behaviour.
